# Modeling astrocyte-neuron interactions in a tripartite synapse

**DOI:** 10.1186/1471-2202-15-S1-P98

**Published:** 2014-07-21

**Authors:** Marja-Leena Linne, Riikka Havela, Aušra Saudargienė, Liam McDaid

**Affiliations:** 1 Computational Neuroscience Research Group, Department of Signal Processing, Tampere University of Technology, Tampere, Finland; 2 Department of Informatics, Vytautas Magnus University, Kaunas, Lithuania; 3 School of Computing and Intelligent Systems, University of Ulster, Northern Ireland

## 

Glial cells (microglia, oligodendrocytes, and especially astrocytes) play a critical role in the central nervous system by affecting in various ways the neuronal single cell level interactions as well as connectivity and communication at the network level, both in the developing and mature brain. Numerous studies (see, e.g., [1-3]) indicate an important modulatory role of astrocytes in brain homeostasis but most specifically in neuronal metabolism, plasticity, and survival. Astrocytes are also known to play an important role in many neurological disorders and neurodegenerative diseases. It is therefore important in the light of recent evidence to assess how the astrocytes interact with neurons, both in situ and in silico. The integration of biological knowledge into computational models is becoming increasingly important to help understand the role of astrocytes both in health and disease. We have previously addressed the role of transmitters and amyloid-beta peptide on calcium signals in rat cortical astrocytes [4]. In this work, we extend the work by using a modified version of the previously developed model [5] for astrocyte-neuron interactions in a tripartite synapse to explore the effects of various pre- and postsynaptic as well as extrasynaptic mechanisms on neuronal activity. We consider extending the model to include various additional mechanisms, such as the role of IP3 receptor function, recycling of neurotransmitters, K+ buffering by the Na+/K+ pump, and retrograde signaling by endocannabinoids. The improved tripartite synapse model for astrocyte-neuron interactions will provide an essential modeling tool for facilitating studies of local network dynamics in the brain. The model may also serve as an important step toward understanding mechanisms behind induction and maintenance of plastic changes in the brain.
